# Identifying and Responding to Lead in Drinking Water in a University Setting

**DOI:** 10.3390/ijerph21050561

**Published:** 2024-04-28

**Authors:** Audrey G. Bousquet, Lauren A. Eaves, Kim Haley, David Catalano, Gregory B. Williams, Hadley J. Hartwell, Catherine Brennan, Rebecca C. Fry

**Affiliations:** 1 Department of Environmental Sciences and Engineering, Gillings School of Global Public Health, University of North Carolina at Chapel Hill, Chapel Hill, NC 27599, USA; aaudrey@live.unc.edu (A.G.B.); laeaves@live.unc.edu (L.A.E.); hadley_hartwell@med.unc.edu (H.J.H.); 2 Institute for Environmental Health Solutions, Gillings School of Global Public Health, University of North Carolina at Chapel Hill, Chapel Hill, NC 27599, USA; 3 Department of Environment, Health and Safety, University of North Carolina at Chapel Hill, Chapel Hill, NC 27599, USA; kphaley@ehs.unc.edu (K.H.); dacatalano@ehs.unc.edu (D.C.); gregory.williams@ehs.unc.edu (G.B.W.); crbrennan@ehs.unc.edu (C.B.); 4 Curriculum in Toxicology and Environmental Medicine, School of Medicine, University of North Carolina at Chapel Hill, Chapel Hill, NC 27599, USA

**Keywords:** lead, drinking water, water sampling, university

## Abstract

Lead is an established neurotoxicant, and it has known associations with adverse neurodevelopmental and reproductive outcomes. Exposure to lead at any level is unsafe, and the United States (US) has enacted various federal and state legislations to regulate lead levels in drinking water in K-12 schools and childcare facilities; however, no regulations exist for higher education settings. Upon the discovery of lead in drinking water fixtures in the University of North Carolina at Chapel Hill (UNC-CH) campus, a cross-campus water testing network and sampling plan was developed and deployed. The campaign was based on the US Environmental Protection Agency’s (EPA) 3Ts (Training, Testing, and Taking Action) guidance. The seven-month campaign involved 5954 tests on 3825 drinking water fixtures across 265 buildings. A total of 502 (8.43%) tests showed lead above the limit of detection (1 part per billion, ppb), which represented 422 (11.03%) fixtures. Fewer than 1.5% of the tests were above the EPA action level for public water systems (15 ppb). In conclusion, systematic testing of all the fixtures across campus was required to identify localized contamination, and each entity in the cross-campus network undertook necessary roles to generate a successful testing campaign. UNC-CH established preventative measures to test drinking water fixtures every three years, which provide a framework for other higher education institutions in responding to lead contamination.

## 1. Introduction

Exposure to lead at any level is unsafe, with adverse neurodevelopmental and reproductive effects documented at levels even below the regulatory standards [1,2,3,4,5,6]. Pregnant individuals, infants, children, and elderly individuals are considered the populations most vulnerable to exposure [1,2,3,4,5,6,7]. In utero exposure to lead is associated with preterm birth and a low birth weight, both of which are linked to several adverse health outcomes throughout the course of life [1,2]. Infants and children exposed to lead have an increased risk of adverse neurobehavioral and cognitive outcomes [4,6]. Older adults exhibit a reduced cognitive function and increased frailty when exposed to lead cumulatively throughout the course of life [5,7]. Despite the particular susceptibility of these populations, the effects of lead exposure transcend health and age status to universally impact individuals.

Other than the global phase-out of leaded gasoline, lead regulations vary greatly worldwide [8]. Consumption of lead-contaminated water is one of the primary sources of lead exposure [9]. The World Health Organization (WHO) has set a guideline value for lead to be no greater than 10 parts per billion (ppb) in drinking water, a threshold many countries have implemented as their benchmark lead level [10]. Despite the actions numerous industrialized countries have taken to reduce exposure, many continue to struggle with lead contamination due to a lack of regulation in unsuspecting settings, including the United States (US) [8].

The US began to regulate lead in the 1970s with federal legislation including, among others, the 1974 Safe Drinking Water Act, the 1988 Lead Contamination Control Act, the 1991 Lead and Copper Rule, and the 2011 Reduction of Lead in Drinking Water Act [11,12,13,14]. As part of the Safe Drinking Water Act, the Environmental Protection Agency (EPA) sets non-enforceable health goals, known as maximum contaminant level goals (MCLGs): the highest level of a contaminant in drinking water where no established or expected adverse health effects exist. The MCLG for lead is zero [15]. Following the Safe Drinking Water Act, the Lead Contamination Control Act established state childhood lead prevention programs, supported by the Centers for Disease Control and Prevention (CDC), and imposed limits on lead concentrations (<8%) in components that come in contact with drinking water in K-12 schools and childcare facilities [12]. Notably, this legislation does not apply to higher education institutions. In 2011, the Reduction of Lead in Drinking Water Act adjusted the definition of “lead-free” by reducing the maximum lead content of plumbing supplies from 8% to 0.25% [14]. Additional regulations to annually test community water systems were implemented by the Lead and Copper Rule, with requirements for systems to be remediated if more than 10% of serviced tap water samples exceed 15 ppb of lead, termed the EPA action level (AL) [15].

In addition to federal regulations, US states also have specific regulations to mitigate lead exposure. In North Carolina (NC), lead regulation and programming include the 1997 Childhood Lead Exposure Control Act, the NC Lead Surveillance System, and the NC Childhood Lead Poisoning Prevention Program (CLPPP) [16,17,18,19]. In 2021, the CLPPP updated the NC lead hazard level for drinking water in childcare and school facilities to be 10 ppb (the WHO guideline level), 5 ppb lower than the previous level [19]. This program also requires facilities to conduct water testing every three years, which does not apply to higher education institutions.

These federal and state lead regulations, among others that manage lead levels in gasoline, paint, and various consumer products, significantly decreased the mean blood lead level (BLL) of populations within the US. Between 1976 and 2016, the mean BLL decreased by 95% among US children aged 1–11 years and by 94% among individuals of childbearing age [20,21]. Still, it is estimated that over 500,000 individuals of childbearing age have a BLL > 5 μg/dL, which was the previous CDC blood lead reference value [20]. As of 2021, the blood lead reference value is now 3.5 μg/dL [20]. Although there have been substantial reductions in BLL, the US population still faces significant health risks due to environmental and occupational lead exposure [22].

While US state and federal regulations are in place for community water systems, schools (K-12), and childcare facilities, there are no such regulations for lead testing in higher education institutions. Thus, drinking water on university campuses is seldom tested, ultimately creating a potential public health concern for students, faculty, and staff. The California State University–Sacramento, University of Michigan–Dearborn, University of Michigan–Flint, Princeton University, and others are among the few universities in the US that have conducted water testing on their campuses [23,24,25,26,27,28,29]. At the international level, the National Taiwan University has also reported testing their campus drinking water for lead [30]. The rarity of water testing is especially a concern for historic universities with buildings constructed before lead regulation went into effect in the 1970s. A spotlight was shone upon the University of North Carolina at Chapel Hill (UNC-CH) in 2022, when elevated levels of lead were detected in campus drinking water fixtures. Upon this discovery, the university developed and deployed a campus-wide comprehensive water testing campaign. In this article, we describe the scope of lead contamination in drinking water at UNC-CH and the action plan taken to remediate the problem. We also review key takeaways and provide recommendations for other higher education institutions.

## 2. Materials and Methods

### 2.1. Timeline of Process and Sampling Plan

**Figure 1:**
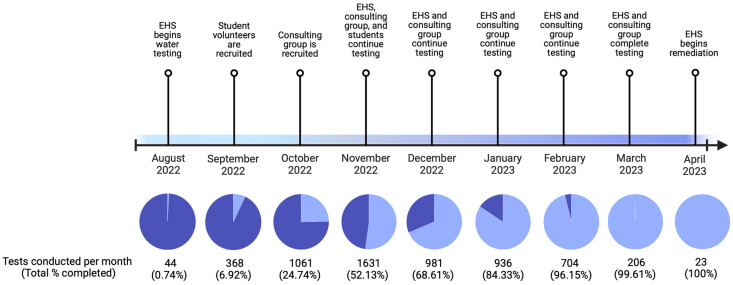
Timeline of water sampling process from discovery of contamination to completion of sampling and start of remediation. Light blue indicates the percent of tests completed out of *n* = 5954.

Figure 1 provides an overview of UNC-CH’s water sampling timeline. In August 2022, lead was identified in samples drawn from fixtures in a historic building on campus. Environment, Health, and Safety (EHS) was alerted and repeat sampling confirmed detectable lead. In response, EHS mobilized a campus-wide network of 11 entities and developed a water-sampling plan based on the EPA’s 3Ts (Training, Testing, and Taking Action) guidance [31]. Due to the number of buildings on campus, a three-phase approach to systematically test all fixtures within each campus building was employed. Phase one focused on fixtures in buildings constructed in or before 1930. Phase two sampled and tested fixtures in buildings constructed between 1930 and 1990. Phase three sampled and tested fixtures in buildings constructed post-1990.

Sampling began with three EHS staff in August 2022. At the end of September 2022, EHS partnered with a professor in the Department of Environmental Sciences and Engineering in the Gillings School of Global Public Health to act as a scientific advisor, whose primary role was to work alongside UNC Communication and provide information on the adverse effects of lead exposure. Together, EHS and the scientific advisor recruited and trained a total of 29 student volunteers in September–November 2022, who conducted additional water sampling. To enable larger-scale testing, in October 2022, UNC-CH hired a consulting group of four individuals. Student volunteer efforts ended in December 2022, while EHS and the consulting group continued and completed sampling and testing in April 2023. EHS continued to remediate and retest fixtures that returned detectable levels of lead.

### 2.2. Sampling Procedures

The sampling plan was developed based on the EPA’s 3Ts guidance [31]. Due to variations in the composition and dimension of each fixture type, the sampling procedure varied between (1) water fountains and bottle fillers and (2) sinks and other fixtures (including ice makers and water dispensers).

For all fixture types, a two-day testing procedure was employed. On day one, water fountains and bottle fillers were flushed for 15 min to remove stagnation. Sinks and other fixtures were flushed for 30 s with the cold tap setting. Post-flushing, fixtures were made inaccessible for use. On day two, water samples were collected immediately after turning on the water source, following an 8 to 18 h stagnation period. For water fountains and bottle fillers, two consecutive samples were taken: The first sequential sample (1SS) collected the first 125 mL, in order to test the water in contact with the outlet/bubbler. The second sequential sample (2SS) collected 250 mL immediately following the 1SS, in order to test the water in contact with the storage tank and inlet strainer. For sinks and other fixtures, one sample of 250 mL was collected using the cold tap setting (“first draw”). Second samples (“second draw”) of 250 mL were collected from sinks and other fixtures on a separate day if the first draw had detectable lead. The first and second draw samples represent water in contact with the faucet or dispenser and connected plumbing.

Water samples were collected in 250 milliliter (mL) high-density polyethylene bottles (Pace Analytical Services, LLC, Oldsmar, FL, USA and Mount Juliet, TN, USA, and ©Eurofins Scientific, Savannah, GA, USA) that were prefilled with 2 mL nitric acid for preservation purposes. Data for each sample (e.g., 4-digit sample number, building name, building floor number, room number, time of collection, fixture type, sample type (1SS, 2SS, first draw, second draw), and lead test results) were collected using ©2022 Veoci Inc developed by Information Technology Systems (ITS) at UNC-CH.

Following sample collection, the fixtures were made inaccessible for use until the lead-testing results became available. If lead was not detected, the fixtures were returned to operational status. If lead was detected, the fixtures were taken out of service, and retesting and remediation ensued.

### 2.3. Campus Water Source

The water in each campus building is sourced from a local lake and reservoir and is treated by the local water treatment plant (Orange Water and Sewer Authority, OWASA) [32]. Water at this facility undergoes various water treatments, including a corrosion control program to prevent lead [32]. Given that the OWASA monitors for lead, it was considered an unlikely source of lead contamination. Thus, EHS focused on possible fixture-related sources of lead (e.g., brass fittings) and, to a lesser extent, building-related sources, such as leaded plumbing components (e.g., backflow preventers).

### 2.4. Lead Analysis

For lead concentration determination, EPA Method 200.8 Determination of Trace Elements in Waters and Wastes by Inductively Coupled Plasma–Mass Spectrometry (ICP-MS) was used [33]. The limit of detection (LOD) for this method was 1 ppb. Water samples collected by EHS were sent to Pace Analytical Services, LLC (Oldsmar, FL, USA, and Mount Juliet, TN, USA). Samples collected by the consulting group were sent to ©Eurofins Scientific (Savannah, GA, USA). Samples were analyzed on a Thermo Scientific™ iCAP™ RQ ICP-MS (Bremen, Germany) in batches as they were collected. To ensure the integrity of each analysis, the quality control measures of EPA method 200.8 were used, which included the assessment of a method blank (water), a laboratory control sample, and two matrix spikes alongside their matrix spike duplicates. SRM 3128 (High Purity Standards, North Charleston, SC, USA) was used as a certified reference material for lead measurements by ICP-MS. No additional analyses for other parameters, such as pH, temperature, or conductivity, were conducted.

### 2.5. Statistical Analysis

All data collected during the water sampling operation were analyzed. The full dataset contained the following information: building, fixture type, sample type (1SS, 2SS, first draw, second draw), and lead concentration (ppb). Using the UNC Facilities Services building database, buildings were matched to their year of construction and year of latest renovation. Values below the LOD (1 ppb) were imputed as the LOD/sqrt(2) [34]. Four relevant standards were utilized in analyzing the distribution of lead: (1) the American Academy of Pediatrics (AAP) hazard reference level (1 ppb), which was also the LOD; (2) the NC hazard level (10 ppb), which was originally established for children under the age of six and is also the WHO recommended guideline value; (3) the EPA AL (15 ppb); and (4) the EPA 3Ts’ recommended 20 ppb level (or 5 µg per 250 mL) for the identification of lead sources at individual outlets [10,15,19,31,35]. For each fixture type and building age group, the number and percentage of samples equal to or above the LOD, 10, 15, and 20 ppb were calculated. Data cleaning and analysis were conducted in R (v 4.2.2).

### 2.6. Remediation

EHS, with assistance from Facilities Services, began remediation in February 2023. Remediation ensued for any fixture that was identified as having a detectable level of lead. The remediation process was as follows: First, EHS determined whether a building-wide or localized contamination concern existed for each building with at least one fixture with detectable lead. Multiple factors were considered in this assessment, including the age, make, model, and prevalence of fixtures with detected lead, compared to those with no lead detected, in one building. Second, fixtures with detectable lead above the EPA AL were entirely replaced. Third, for fixtures with results between the LOD and EPA AL, EHS performed an iterative process to pinpoint the fixture component contributing to the detectable lead. This process varied by fixture type.

For drinking fountains, detectable lead in the 1SS indicated that the potential source was the outlet (bubbler). Detectable lead in the 2SS indicated that the potential source was the storage tank or inlet strainer. Based on the results, the remediation of drinking fountains began with the cleaning or replacement of the outlet and/or inlet strainer. Following this initial remediation step, fixtures were retested and placed back in service if the result was below the LOD. If samples taken after the cleaning or replacement of fixture components continued to return detectable lead, water filters were installed. For sinks with detectable lead at levels below the EPA AL, aerators (inlet screens) were replaced, followed by retesting. If lead was still detected, the faucet was replaced, and if lead remained detectable after faucet replacement, filters were installed until the result was below the LOD.

## 3. Results

### 3.1. Scope of Lead Contamination on UNC-CH Campus

From 22 August 2022 to 5 April 2023, 5954 tests were conducted on 3825 fixtures in 265 buildings (Table 1). A total of 1616 1SS and 1615 2SS tests were conducted on water fountains, and 487 1SS and 486 2SS tests were conducted on bottle filters. A total of 1572 first draw and 27 second draw tests were completed on sinks. For the remaining fixture types (ice makers, water dispensers, etc.), 149 first draw tests and two second draw tests were completed. Of the 5954 tests conducted, 502 (8.43%) were above the LOD of 1 ppb (the AAP hazard reference level). A total of 83 (1.39%) tests were above 10 ppb (the NC hazard level, WHO guideline value), 61 (1.02%) were above 15 ppb (the EPA AL level), and 50 (0.84%) were above 20 ppb, which is the EPA recommended threshold for the 3Ts plan (Table 1). Of 3825 fixtures, 422 (11.03%) had at least one sample above the LOD. A total of 72 (1.88%) fixtures had at least one sample above 10 ppb, 52 (1.35%) had at least one sample above 15 ppb, and 42 (1.10%) had at least one sample above 20 ppb. Of 265 buildings, 150 (56.60%) had at least one sample above the LOD, 42 (15.85%) had at least one sample above 10 ppb, 30 (11.32%) had at least one sample above 15 ppb, and 25 (9.43%) had at least one sample above 20 ppb.

**Figure 2:**
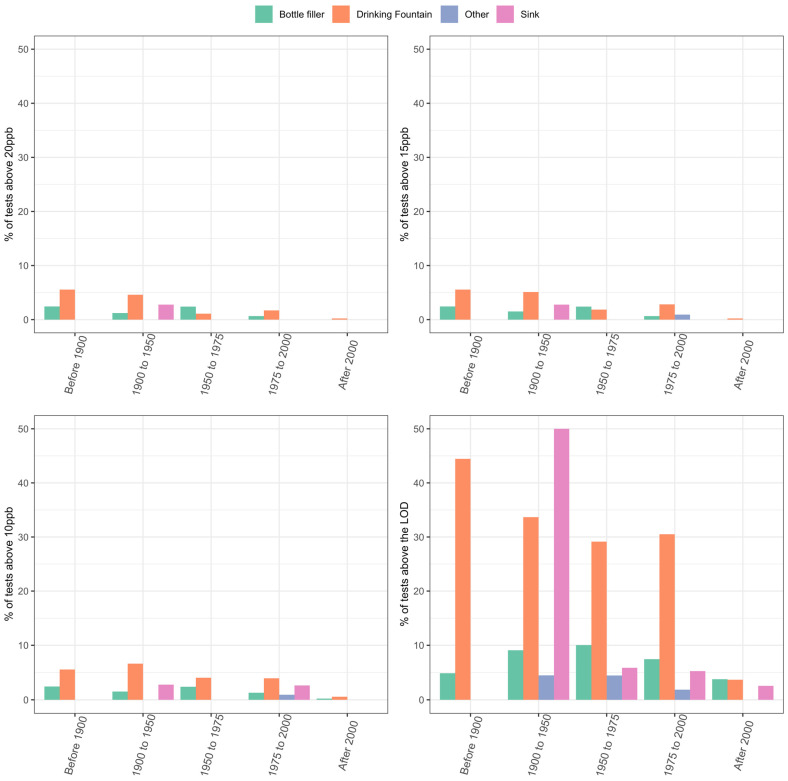
Percentage of tests conducted that were equal to or above 20 ppb, 15 ppb, or 10 ppb or above the limit of detection (LOD), 1 ppb, by year of building construction and type of fixture.

Samples from sinks had the highest prevalence of detected lead. Among first draws, sinks had detectable lead in 15.71% of samples, compared to 7.74% and 2.87% for drinking water fountains and bottle fillers, respectively (Table 1 and Figure 2). Furthermore, 2.35% of first draws from sinks were above 10 ppb, while 1.30% and <1% of samples were above 10 ppb from drinking water fountains and bottle fillers, respectively (Table 1 and Figure 2).

The prevalence of detectable lead increased based on a building’s age from 1900 to 1975, with buildings constructed before 1900 having 10.53% of samples with detectable lead, buildings between 1900 and 1950 having 12.33% detectable lead, and those constructed between 1950 and 1975 having 11.72% detectable lead (Table 2 and Figure 2). In buildings constructed after 1975, detectable lead decreased. Buildings constructed between 1975 and 2000 had 9.21% of the samples with detectable lead, and those constructed after 2000 had 2.59% detectable lead.

**Figure A1:**
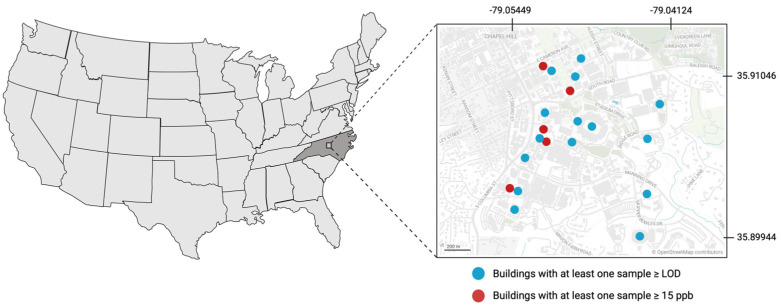
UNC-CH is located in central North Carolina, USA. The magnified campus map depicts the location of the top 20 priority buildings, based on the percentage of samples above the LOD. Buildings labeled in blue had at least one water sample with lead levels equal to or above 1 ppb (the LOD). Buildings labeled in red had at least one sample with lead levels equal to or above 15 ppb.

To identify key buildings of concern (priority buildings), analyses focused on those with over 10 samples collected and ranked buildings by the percentage of 1SS samples with detectable lead from drinking water fountains or bottle fillers (Appendix A, Table A1). In the building with the highest prevalence of detectable lead, the percentage of tests above the LOD was 37.50%, and the percentage of tests above 10 ppb, 15 ppb, and 20 ppb, were 20.83%, 16.67%, and 8.33%, respectively. The maximum level of lead detected in this building was 180 ppb. Of the 20 priority buildings identified, 13 (65%) were built before 1975. The final two priority buildings each had <10% of samples returning detectable lead levels. Collectively, there were no discernable geospatial patterns among the 20 buildings (Appendix A, Figure A1).

Overall, the maximum concentration of lead detected in all samples was 1100 ppb, which originated from a 2SS collected from a drinking fountain in a building constructed in 1969 (Table 1 and Table 2). The sample type with the highest mean concentration of lead, 2.61 ppb, was 2SS (Appendix A, Table A2). The mean and median lead concentrations for all sample types were 1.69 ppb and 0.71 ppb, respectively (Appendix A, Table A2). In comparison to other universities, UNC-CH’s water-testing campaign collected five times the number of samples as the next highest comparable institution (e.g., 5954 tests conducted versus 1135 tests). UNC-CH also had the lowest number of samples exceeding the LOD (8.43%) relative to the total number of tests conducted (Table 3).

### 3.2. Implementation of Action Plan

#### 3.2.1. Mobilization of Campus Network

**Figure A2:**
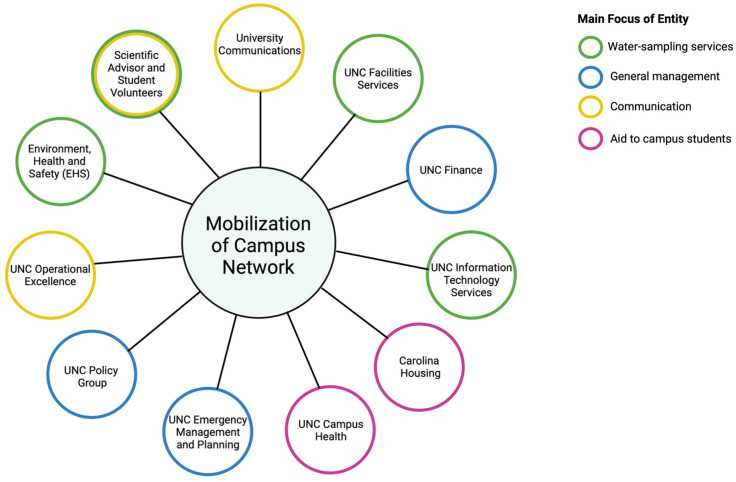
Eleven campus entities were mobilized to form the campus-wide network required to oversee the water-testing campaign, the communication of the findings, and remediation efforts.

A network of 11 campus entities was established to address the lead contamination (Appendix A, Figure A2). The entities involved included the following: (1) EHS supervised the water sampling of all buildings across campus, including conducting in-house sampling and managing the student volunteers and consulting group; the University Employee Occupational Health Clinic offered medical advice and free BLL testing to employees. (2) University Communications managed internal (e.g., campus emails) and external communications (e.g., media interviews). (3) UNC Facilities Services oversaw the sourcing, purchasing, and installation of new fixtures as needed, as well as sourcing and deploying water bottles throughout testing. Building managers conveyed lead results to their building occupants. (4) UNC Finance procured contracts for additional testing personnel and supplies, such as temporary water dispensers and bottled water. (5) UNC Information Technology Services developed workflows for data capture of the sampling. (6) Carolina Housing coordinated remediation efforts with EHS and Facilities Services in residence halls, which were designated as highest urgency for testing and remediation. (7) UNC Campus Health provided medical advice and offered free BLL testing to students and post-doctoral fellows. (8) UNC Emergency Management and Planning facilitated cross-campus meetings for all involved entities, coordinated data collection, and published internal status update reports. (9) The UNC Policy Group approved strategic objectives brought up by the Operations Group. (10) UNC Gillings School of Global Public Health provided a scientific advisor, communications advisors, and student volunteers. (11) The UNC Operational Excellence unit served as a liaison to the Chancellor and Provost. EHS collaborated with the OWASA, a non-campus partner, to interpret some of the sampling results. From September to November 2022, the point person from each entity met twice per week. Throughout December 2022, this network met once per week. In spring 2023, the frequency of meetings declined to once every two weeks, followed by once per month.

#### 3.2.2. Dissemination of Information

Two main approaches were utilized to communicate the lead-testing results. First, building managers were notified of the results, and potential retesting and remediation efforts, directly by EHS via email. The managers distributed the testing results to their building occupants. Second, public-facing outlets were leveraged, including (1) a webpage dedicated to publishing the testing results and (2) updates on the EHS’ X™ account.

#### 3.2.3. Economic Implications

The overall cost of the water-sampling operation was USD 553,377. The breakdown of costs was as follows: USD 2216 for testing services for water samples collected by EHS and student volunteer staff, USD 24,570 in bottled water refills, USD 5303 for filter installation in existing fixtures, USD 54,119 for water cooler replacement, repair, and maintenance, USD 11,094 for signage and stickers, USD 202 for drinkware supplies, and USD 455,873 for water sampling (including the cost of the consulting group). Student volunteers were compensated with a USD 25 gift card (totaling: USD 725) not included in the total cost. Remediation costs and costs to support staff efforts on this project are not included in this calculation.

#### 3.2.4. Remediation Results

As of April 2024, EHS had remediated 417 (98.81%) of fixtures that returned lead levels above the LOD (*n* = 422). All fixtures with lead concentrations above the EPA AL were replaced entirely, while fixtures with lead levels between the LOD and EPA AL underwent component-specific cleaning and replacement. Water filters were installed on fixtures that continued to return detectable lead following remediation.

## 4. Discussion

Exposure to lead is associated with various adverse health effects, yet regulation of this toxic metal varies across the globe [1,2,3,4,5,6,7]. Most industrialized countries have regulations established to reduce lead usage, whereas other low- and lower-middle-income countries have few standards in place [8]. In the US, there are numerous federal and state regulations for lead in drinking water; however, no laws specifically apply to monitoring lead in higher education institutions. There are approximately 4000 degree-granting postsecondary schools within the US with 16 million students [36,37]. At a global scale, approximately 200 million students are enrolled in postsecondary schools [38]. Therefore, it is crucial to address concerns regarding environmental safety in higher education institutions. After lead was detected on UNC-CH’s campus in 2022, a systematic, seven-month water-testing campaign was launched. There were three major findings from the current study. First, lead levels ranged up to 1100 ppb in drinking water samples on campus. Second, buildings constructed prior to 1975 had higher lead levels than newer buildings. Third, the strategy UNC-CH employed was successful at addressing the contamination issue, providing a framework for other institutions.

The UNC-CH testing campaign comprised 5954 tests on 3825 fixtures across 265 buildings. Collectively, 8.43% of tests and 11.03% of fixtures were above the LOD (1 ppb). Fewer than 1.02% of tests and 1.35% of fixtures were above the EPA AL (15 ppb). The maximum concentration of lead detected in all samples was 1100 ppb. Despite some samples having elevated lead levels, the mean concentration of all samples collected was 1.69 ppb, which is just above the LOD. The median concentration of lead was 0.71 ppb, which is below the LOD. When compared to published or publicly available water-testing initiatives at higher education institutions, UNC-CH executed a robust campaign testing ~6000 samples. The systematic plan to test all the fixtures in every building on campus resulted in five times the number of samples as the next highest comparable institution, a difference that was not influenced by institution size. Of note, UNC-CH had substantially fewer samples exceeding the LOD when compared to the total number of tests conducted at other institutions [23,24,25,26,27,28,29,30]. These results build upon research documenting lead exposure concerns in NC, as well as throughout the US and Canada in the home setting, particularly among private well water users, as well as in earlier education settings [39,40,41,42,43].

The results of the present study highlighted that water samples collected from sinks, particularly in buildings constructed between 1900 and 1950, had the highest lead prevalence. This could be attributed to their infrequent usage and therefore increased risk of corrosion. This is in comparison to regularly used water fountains and bottle fillers. In general, buildings constructed before 1975 had a higher lead prevalence than newer buildings which is a correlation consistent with the onset of lead regulations in the 1970s. Of the 20 buildings with the highest number of tests with lead levels above the LOD, 13 (65%) were constructed prior to 1975. The highest-priority building was constructed in 1969. This building had 37.50% of samples collected from its fixtures returning lead levels above the LOD. Supporting the findings of the present study, in NC childcare facilities, building age was a significant predictor of higher lead levels [42].

Overall, this campaign was successful in identifying, assessing, mitigating, and remediating lead contamination. This success can be attributed to four key strengths, outlined as recommendations below. These strengths can provide guidance to institutions that may face similar challenges. The first recommendation is to engage and coordinate entities broadly across campus and seek external support. Eleven different campus entities were mobilized into a cohesive network that directed all the steps of the campaign, including water sampling, remediation, the dissemination of results, and communication to the campus community and public. The efforts of this network were essential to the success of UNC-CH’s testing campaign, as each entity held a crucial and unique role. When the scope of the sampling needs was fully realized, an external consulting group was hired to expedite the sampling efforts to the rate required. After the external support commenced, the rate of water sampling accelerated by a factor of three, and the overall campaign timeline was shortened significantly. The second recommendation is to systematically test all the fixtures in all buildings in response to the identification of localized lead contamination. Research conducted in school settings underscores that taking samples from select fixtures within a building can miss critical lead sources in specific fixtures; thus, all fixtures must be tested [44]. The third recommendation is to provide transparent communication about the testing efforts to the campus community and public. This was essential to mitigate fear and confusion. The fourth recommendation is to remediate fixtures with detectable lead by carefully considering potential sources. Lead can be released into water proximal to the point of use (i.e., in the faucet fixtures), or it can be released from a more distant source, such as corrosion from lead service lines, each requiring different interventions [42,45]. Complicating matters further, currently available “lead-free” fixtures have been found to leech lead at levels above 1 ppb (the LOD that initiated remediation) [46]. Moreover, research has documented that the mitigation approach of daily first flushing does not always reduce lead levels below regulatory standards [44].

UNC-CH is currently in the process of remediating fixtures with detectable lead by implementing a stepwise approach for each building and fixture. As of April 2024, EHS has remediated 417 (98.81%) fixtures. Further analysis, including the total cost of the campaign, will be conducted once the remediation efforts are completed. To implement ongoing preventative measures, routine water sampling of all fixtures in UNC-CH campus buildings built before 2014 will occur every three years under a staggered action plan. This testing frequency mirrors the regulation in 15A NCAC 18A. 2816 Lead Poisoning Hazards in Childcare Centers [40]. Fixtures that return detectable lead will be remediated and retested within one year, to ensure efforts maintain non-detectable levels. EHS and Facilities Services will also verify that plumbing components and filters meet the NSF/ANSI 372 and 53 standards for lead content and reduction, respectively [41,42].

While this study is among the largest systematic testing initiatives of lead in a higher education institution, it is not without limitations. Replicate samples were only collected for select fixtures due to the broad scope of the campaign. Data confidence would be enhanced with increased sample replicates. Samples were analyzed at more than one laboratory, potentially influencing data precision.

## 5. Conclusions

In conclusion, the discovery of the presence of lead in drinking water on UNC-CH’s campus resulted in a testing and remediation campaign that can serve as a framework for other higher education institutions in the US and beyond. Given the lack of regulation regarding water testing in higher education settings, other post-secondary institutions may have comparable challenges to address. Lead contamination is a preventable issue, provided that effective regulatory measures are in place [9]. Ultimately, lead testing will protect the health of millions of students and likely hundreds of thousands of staff and faculty who live and work at higher education institutions.

## Figures and Tables

**Table 1:** Summary of number of tests conducted, number and percentage of tests above LOD, 10 ppb, 15 ppb, and 20 ppb by different fixture type.

Fixture Type	Sample Type	Number of Tests	Number (%) of Tests above LOD	Number (%) of Tests above 10 ppb	Number (%) of Tests above 15 ppb	Number (%) of Tests above 20 ppb	Maximum (ppb)
Drinking fountain	1SS	1616	125 (7.74)	21 (1.3)	16 (0.99)	13 (0.80)	515.00
	2SS	1615	97 (6.01)	20 (1.24)	20 (1.24)	18 (1.24)	1100.00
Bottle filler	1SS	487	14 (2.87)	1 (0.21)	1 (0.21)	0 (0)	16.00
	2SS	486	9 (1.85)	0 (0)	0 (0)	0 (0)	4.60
Sink	1st	1572	247 (15.71)	37 (2.35)	23 (1.46)	18 (1.15)	96.30
	2nd	27	1 (3.7)	0 (0)	0 (0)	0 (0)	5.10
Other *	1st	149	8 (5.37)	2 (1.34)	1 (0.67)	1 (0.67)	70.10
	2nd	2	1 (50)	0 (0)	0 (0)	0 (0)	5.80
All Fixtures	1SS	2103	139 (6.61)	22 (1.05)	17 (0.81)	13 (0.62)	515.00
	2SS	2101	106 (5.05)	22 (1.05)	20 (0.95)	18 (0.86)	1100.00
	1st	1721	255 (14.82)	39 (2.27)	24 (1.39)	19 (1.1)	96.30
	2nd	29	2 (6.9)	0 (0)	0 (0)	0 (0)	5.80
	All sample types	5954	502 (8.43)	83 (1.39)	61 (1.02)	50 (0.84)	1100.00

* Other includes ice makers, water dispensers, and water filtering systems. 1SS = first sequential sample, 2SS = second sequential sample. 1st = first draw, 2nd = second draw conducted at a later date if the first draw was above the LOD. 1SS and 2SS samples were collected for drinking water fountains, first and second draws were collected for all other fixture types.

**Table 2:** Summary of number of tests conducted, number and percentage of tests above LOD, 10 ppb, 15 ppb, and 20 ppb by building age. Note, 4 buildings did not have building construction date data available, therefore this table includes the 5879 tests for which building data were available.

Building Year of Construction	Number of Buildings	Sample Type	Number of Tests	Number (%) of Tests above LOD	Number (%) of Tests above 10 ppb	Number (%) of Tests above 15 ppb	Number (%) of Tests above 20 ppb	Maximum (ppb)
Before 1900	10	1SS	46	2 (4.35)	1 (2.17)	1 (2.17)	1 (2.17)	515.00
		2SS	46	2 (4.35)	1 (2.17)	1 (2.17)	1 (2.17)	254.00
		1st	19	8 (42.11)	1 (5.26)	1 (5.26)	1 (5.26)	31.30
		2nd	3	0 (0)	0 (0)	0 (0)	0 (0)	0.71
		All	114	12 (10.53)	3 (2.63)	3 (2.63)	2 (1.75)	515.00
1900 to 1950	66	1SS	419	34 (8.11)	4 (0.95)	4 (0.95)	3 (0.72)	74.00
		2SS	419	30 (7.16)	5 (1.19)	5 (1.19)	4 (0.95)	190.00
		1st	232	68 (29.31)	14 (6.03)	11 (4.74)	10 (4.31)	96.30
		2nd	9	1 (11.11)	0 (0)	0 (0)	0 (0)	5.80
		All	1079	133 (12.33)	23 (2.13)	20 (1.85)	10 (0.93)	190.00
1950 to 1975	54	1SS	637	54 (8.48)	11 (1.73)	9 (1.41)	7 (1.1)	180.00
		2SS	637	50 (7.85)	11 (1.73)	11 (1.73)	11 (1.73)	1100.00
		1st	305	81 (26.56)	11 (3.61)	5 (1.64)	3 (0.98)	77.00
		2nd	8	1 (12.5)	0 (0)	0 (0)	0 (0)	5.10
		All	1587	186 (11.72)	33 (2.08)	25 (1.58)	3 (0.19)	1100.00
1975 to 2000	51	1SS	416	25 (6.01)	5 (1.2)	3 (0.72)	2 (0.48)	220.00
		2SS	415	16 (3.86)	2 (0.48)	2 (0.48)	2 (0.48)	662.00
		1st	215	56 (26.05)	8 (3.72)	5 (2.33)	3 (1.4)	31.90
		2nd	7	0 (0)	0 (0)	0 (0)	0 (0)	0.71
		All	1053	97 (9.21)	15 (1.42)	10 (0.95)	3 (0.28)	662.00
After 2000	57	1SS	555	17 (3.06)	1 (0.18)	0 (0)	0 (0)	10.00
		2SS	554	2 (0.36)	0 (0)	0 (0)	0 (0)	1.60
		1st	935	34 (3.64)	5 (0.53)	2 (0.21)	2 (0.21)	54.00
		2nd	2	0 (0)	0 (0)	0 (0)	0 (0)	0.71
		All	2046	53 (2.59)	6 (0.29)	2 (0.1)	2 (0.1)	54.00

1SS = first sequential sample, 2SS = second sequential sample. 1st = first draw, 2nd = second draw conducted at a later date if the first draw was above the LOD. 1SS and 2SS samples were collected for drinking water fountains, first and second draws were collected for all other fixture types.

**Table 3:** Lead concentration data from UNC-CH and comparable institutions that conducted lead testing in drinking water on their campus.

University	Number of Tests Conducted	Number (%) of Tests above LOD	Number (%) of Tests above 15 ppb	Maximum (ppb)
UNC-CH	5954	502 (8.43%)	61 (1.02%)	1100
California State University–Sacramento ([23])	1135	500 (44.05%)	67 (5.90%)	400
University of Michigan–Dearborn [24]	215	19 (8.84%)	1 (0.46%)	23
University of Michigan–Flint * [25]	72	9 (12.50%)	0	14
Princeton University [26]	77	Data unavailable	0	Data unavailable
University of Oregon [27]	136	87 (63.97%)	1 (0.73%)	18.8
Binghamton University [28]	82	23 (28.05%)	0	11
Wright State University [29]	60	11 (18.33%) ^+^	0	7.7
National Taiwan University [30]	290	290 (100%)	24 (8.27%)	62.6

* Most recent results were published in August 2023; however, the most recent data that are obtainable are from August 2022. ^+^ LOD was 2 ppb.

## Data Availability

The full dataset presented in this study can be made available upon direct request to the authors.

## Appendix A

**Table A1:** Top twenty buildings of concern, according to the percentage of tests above the LOD in 1SS samples in drinking water fountains. Restricted to buildings with over 10 samples collected.

Building	Number of Tests	Number (%) of Tests above LOD	Number (%) of Tests above 10 ppb	Number (%) of Tests above 15 ppb	Number (%) of Tests above 20 ppb	Maximum (ppb)	Year of Building Construction	Year of Last Building Renovation
Brinkhous–Bullitt Building	24	9 (37.5)	5 (20.83)	4 (16.67)	2 (8.33)	180	1969	2009
Carrington Hall	27	10 (37.04)	4 (14.81)	3 (11.11)	3 (11.11)	69.6	1969	2005
Hanes Hall	15	3 (20)	0 (0)	0 (0)	0 (0)	3	1950	2008
Hinton James Residence Hall	20	4 (20)	0 (0)	0 (0)	0 (0)	2.9	1966	2009
Dean Smith Center	16	3 (18.75)	1 (6.25)	0 (0)	0 (0)	11	1980	2003
Memorial Hall	11	2 (18.18)	1 (9.09)	1 (9.09)	1 (9.09)	74	1931	2005
Taylor Campus Health Building	11	2 (18.18)	0 (0)	0 (0)	0 (0)	2.6	1975	2005
Wilson Hall	17	3 (17.65)	0 (0)	0 (0)	0 (0)	4.3	1939	2006
Kenan Stadium	18	3 (16.67)	0 (0)	0 (0)	0 (0)	1.3	1926	2009
Isaac M. Taylor Hall	13	2 (15.38)	1 (7.69)	1 (7.69)	1 (7.69)	138	1968	2015
Koury Oral Health Building	13	2 (15.38)	0 (0)	0 (0)	0 (0)	2.2	2008	NA
Hamilton Hall	20	3 (15)	0 (0)	0 (0)	0 (0)	9.6	1968	NA
Wilson Library	20	3 (15)	1 (5)	1 (5)	0 (0)	15.8	1928	NA
Genetic Medicine Research Building	21	3 (14.29)	0 (0)	0 (0)	0 (0)	2	2005	NA
Marsico Hall	31	4 (12.9)	0 (0)	0 (0)	0 (0)	7	2009	NA
Kenan Football Center	16	2 (12.5)	0 (0)	0 (0)	0 (0)	1.6	1997	2010
Van Hecke–Wattach Hall	16	2 (12.5)	0 (0)	0 (0)	0 (0)	1	1966	NA
Bondurant Hall	19	2 (10.53)	0 (0)	0 (0)	0 (0)	7.4	1960	2006
Boshamer Stadium	11	1 (9.09)	0 (0)	0 (0)	0 (0)	3.7	2007	NA
Greenlaw Hall	11	1 (9.09)	0 (0)	0 (0)	0 (0)	1.1	1966	2007

**Table A2:** Summary of number of tests conducted, number and percentage of tests above LOD, 10 ppb, 15 ppb, and 20 ppb, and mean and median lead concentration by sample type.

Sample Type	Number of Tests	Number (%) of Tests above LOD	Number (%) of Tests above 10 ppb	Number (%) of Tests above 15 ppb	Number (%) of Tests above 20 ppb	Mean (ppb)	Median (ppb)	Maximum (ppb)
1SS	2103	139 (6.61%)	22 (1.05%)	17 (0.81%)	13 (0.62%)	1.58	0.71	515
2SS	2101	106 (5.04%)	22 (1.05%)	20 (0.95%)	18 (0.86%)	2.61	0.71	1100
1st draw	1721	255 (14.82%)	39 (2.27%)	24 (1.39%)	19 (1.10%)	1.52	0.71	93.6
2nd draw	29	2 (6.89%)	0	0	0	1.03	0.71	5.8
All samples	5954	502 (8.43%)	83 (1.39%)	61 (1.02%)	50 (0.84%)	1.69	0.71	1100
